# Challenges and Opportunities of Mass Vaccination Centers in COVID-19 Times: A Rapid Review of Literature

**DOI:** 10.3390/vaccines9060574

**Published:** 2021-06-01

**Authors:** Vincenza Gianfredi, Flavia Pennisi, Alessandra Lume, Giovanni Emanuele Ricciardi, Massimo Minerva, Matteo Riccò, Anna Odone, Carlo Signorelli

**Affiliations:** 1 School of Medicine, Vita-Salute San Raffaele University, 20132 Milan, Italy; pennisi.flavia@hsr.it (F.P.); lume.alessandra@hsr.it (A.L.); ricciardi.giovanni@hsr.it (G.E.R.); minerva.massimo@hsr.it (M.M.); signorelli.carlo@hsr.it (C.S.); 2 Care and Public Health Research Institute (CAPHRI), Maastricht University, 6211 Maastricht, The Netherlands; 3 AUSL-IRCCS di Reggio Emilia, Servizio di Prevenzione e Sicurezza Negli Ambienti di Lavoro (SPSAL), Via Amendola n.2, 42122 Reggio Emilia, Italy; matteo.ricco@ausl.re.it; 4 Department of Public Health, Experimental and Forensic Medicine, University of Pavia, 27100 Pavia, Italy; anna.odone@unipr.it

**Keywords:** health planning organizations, mass vaccination, vaccines, COVID-19, rapid review

## Abstract

A mass vaccination center is a location, normally used for nonhealthcare activities, set up for high-volume and high-speed vaccinations during infectious disease emergencies. The high contagiousness and mortality of COVID-19 and the complete lack of population immunity posed an extraordinary threat for global health. The aim of our research was to collect and review previous experiences on mass vaccination centers. On 4 April 2021, we developed a rapid review searching four electronic databases: PubMed/Medline, Scopus, EMBASE, Google Scholar and medRxiv. From a total of 2312 papers, 15 of them were included in the current review. Among them, only one article described a COVID-19 vaccination center; all of the others referred to other vaccinations, in particular influenza. The majority were conducted in the United States, and were simulations or single-day experiences to practice a mass vaccination after bioterrorist attacks. Indeed, all of them were published after September 11 attacks. Regarding staff, timing and performance, the data were highly heterogenous. Several studies used as a model the Center for Disease Control and Prevention guidelines. Results highlighted the differences around the definition, layout and management of a mass vaccination center, but some aspects can be considered as a core aspect. In light of this, we suggested a potential definition. The current review answers to the urgency of organizing a mass vaccination center during the COVID-19 pandemic, highlighting the most important organizational aspects that should be considered in the planning.

## 1. Introduction

At the end of 2019, a novel highly infectious coronavirus, called SARS-CoV-2, emerged in the city of Wuhan (China), causing an outbreak of unknown viral pneumonia. Because of the high contagiousness and mortality, its global spread, the absence of effective drugs and the lack of population immunity, SARS-CoV-2 has rapidly become a global threat [1,2,3,4], culminating in the announcement of a pandemic by the World Health Organization (WHO) on 11 March 2020 [5,6]. With preventive measures limited to nonpharmaceutical interventions (NPI) (i.e., social distancing, extensive lockdown, etc.), of various efficacy and high social costs [7], the development of COVID-19 vaccines has become a globally shared priority, and research pathways were accelerated by pharmaceutical companies and research institutes through the strong support of central governments. Eventually, various types of vaccines have been developed, ranging from more conventional formulates based on SARS-CoV-2 subunits and/or proteins, live-attenuated and inactivated viruses, replicating and nonreplicating viral vectors, virus-like particles, and cell-based vaccines, to the more innovative mRNA/DNA based vaccines [8]. To date, two mRNA vaccine formulates, and two vaccines based on nonreplicating viral vectors have been licensed to emergency use in most high-income countries, making the vaccine available to vaccination campaigns.

Up to 27 April 2021, around 1 billion vaccine doses have been administered worldwide, equal to 13 doses for every 100 people [9]. Vaccination of the world base population is considered the most promising but also the most challenging approach because it requires the safe and effective delivery of millions of vaccine shots in the shortest period of time, while also avoiding health inequalities. The final aim of mass vaccination is to accelerate disease control through a rapid increase in vaccination coverage, achieving immunity levels essential to meet international goals for mortality reduction, and eventually allowing the ease of NPI [10]. To meet this goal, vaccines have to be made available all over the world, but this unprecedented production may fail in its goal without appropriate distribution and delivery of vaccines in targeted populations. In such a setting, mass vaccination centers (MVCs) are fundamental in minimizing the time required to vaccinate the highest number of people [10]. Due to the unique extent of this mass vaccination campaign, it is frequently held in nontraditional or temporary settings, such as in parking lots or large indoor spaces.

Even though mass vaccination campaigns have been a common element of communicable disease control programs (e.g., H1N1 influenza during the early 1970s and then during the winter season of 2008; smallpox; poliomyelitis, and typhoid fever) in both low-middle and high-income countries worldwide—and the Centers for Disease Control and Preventions (CDC) have issued specific guidelines on how to set up mass vaccination clinics for H1N1 campaigns [11]—no clear information has been previously collected and systematically appraised in medical literature. Moreover, the working definition is often inconsistent because it increasingly emerges while health authorities all around the world are implementing MCVs for COVID-19 vaccines. For example, during spring 2021, EuroDisney Paris was temporarily converted into an MVC with a potential capability of 1000 doses/day, while the Health Ministry of Quebec in its guidelines specifically targeted 2500 doses/day, and during May 2021, MVCs from Italian high and medium-sized cities were able to immunize over 4000 subjects in a single day [12,13,14,15]. 

Moreover, it should be considered that MVCs for COVID-19 vaccination is the first mass vaccination of the modern era. In light of the above considerations, we performed a rapid review, of both scientific and grey literature sources, collecting and summarizing the past experiences of the MVCs in terms of buildings, staff, and time requirements, as well as for organizational programs needed. Moreover, based on retrieved information, we suggested a potential definition of a mass vaccination center. Lastly, our ultimate goal was to critically appraise the available evidence with the final aim of timely and efficiently informing policy makers in the organization of MVCs in different settings.

## 2. Materials and Methods

We developed a rapid review using the World Health Organization (WHO) guidelines, “Rapid reviews to strengthen health policy and systems: a practical guide” [16], and the Preferred Reporting Items for Systematic Reviews and Meta-Analyses (PRISMA) guidelines for reporting [17]. A standardized protocol identifying the research question, the search strategy, inclusion and exclusion criteria was developed and shared within the research team and fully approved before starting the review.

### 2.1. Search Strategy and Data Sources

Studies were retrieved by searching four electronic databases: PubMed/Medline, Scopus and Excerpta Medica database (EMBASE) to screen the scientific literature; Google Scholar and medRxiv to identify eligible documents from the grey literature. Additionally, we consulted professionals involved in the prompting of MVC and screened reference lists of the included articles in order to collect any other potentially relevant material. The literature search was carried out on the 4 April 2021 and it was developed based on a combination of keywords related to MVCs (and similar) and management (and similar), including both MeSH terms and free text words. The search strategy was firstly developed in PubMed/Medline and then adapted to the other databases. Keywords were logically combined with the Boolean operators “AND” and “OR”. The full search strategy is available in Table S1.

### 2.2. Inclusion and Exclusion Criteria

Only original papers written in English and with full text available were included. Due to the aim of this review, we chose articles describing the characteristics of organization and implementation of MVCs, both in real world examples and computer simulations. In this review, we considered the following working definition for a mass vaccination center: points of massive dispensing of vaccines to a large share of the general population in response to an outbreak of a contagious disease. Moreover, any type of vaccine administered in mass vaccination campaigns was considered eligible. Indeed, we did not exclusively restrict our search to anti-COVID-19 vaccination. The reason behind this choice was mainly because, due to the novelty of the topic, we expected to find very few studies specifically referring to COVID-19. On the contrary, collected experiences regarding other vaccination campaigns could still provide useful and interesting suggestions. Moreover, in order to be considered eligible, articles should provide information regarding the organization, preparation and implementation of a mass vaccination center. In our research, no time filter was applied. 

### 2.3. Study Selection and Data Extraction

Articles were first assessed based on title and abstract, and only eligible articles were evaluated in full by the researchers (A.L., G.E.R., M.M.). Data extraction was performed using a pre-piloted spreadsheet elaborated in Microsoft Excel^®^ for Windows (Microsoft Corporation, Redmond, WA, USA). To standardize data extraction, a predefined spreadsheet was prepared by the team. Three authors performed data extraction (A.L., G.E.R., M.M.), revised and supervised by a fourth (senior) researcher (V.G.). Several qualitative and quantitative data were extracted from the original studies. Qualitative data recorded included the name of the first author, year of study, country, type of vaccine, preparation needs, layout. Quantitative data extracted included dimension of the mass vaccination center, presence of pharmacy room, restrooms, staffing, medical procedures, time and performance measures.

## 3. Results

### 3.1. Literature Search

**Figure 1:**
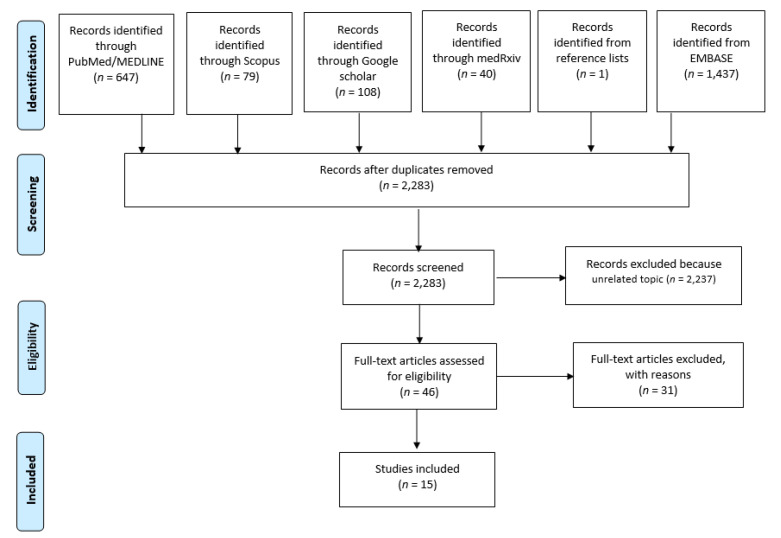
Flow diagram of the selection process.

A total of 2312 articles were initially retrieved by the literature search (Figure 1). After duplicates removal, 2283 articles were left for the title–abstract screening. Based on the title–abstract, 2237 articles were excluded, while the remaining 46 were screened by reading the full-text. In the second screening step, 31 articles were eliminated, and exclusion reasons are listed in Table S2 [18,19,20,21,22,23,24,25,26,27,28,29,30,31,32,33,34,35,36,37,38,39,40,41,42,43,44,45,46,47,48]. In brief, the full texts of four articles were not available despite many efforts to find them [18,19,20,21]. Nine articles did not provide details about the organization of an MVC [22,23,24,25,26,27,28,29,30], rather discussing challenges of mass anti-COVID-19 vaccination [23] or electronic systems used to monitor vaccine reactions and side effects [26]; or the time currently spent by primary care personnel in vaccinating [30]. At the end of the selection process, 15 articles were included in the current review [49,50,51,52,53,54,55,56,57,58,59,60,61,62,63], of which one article was identified screening the reference list [49].

### 3.2. Characteristics of Included Studies

Among the 15 retrieved articles, 14 were published on peer-reviewed scientific journals [49,50,51,52,53,54,55,56,57,58,59,60,61,63]; the remaining one was a newspaper article [62]. All but two articles [58,63] reported on experiences based in the USA, for a timeframe ranging from 2003 [50] to 2021 [62]. Interestingly, a significant share of the retrieved articles followed the great attention posed by US Institutions, after the September 11 attacks in 2001, to develop standard procedures in case of a bioterrorist attack or pandemic flu. Regarding the 13 USA-based articles, nine reported on seasonal influenza and H1N1 vaccination [51,53,54,55,56,57,59,60,61], two on smallpox vaccination [49,50], and only one on the anti-SARS-CoV-2 vaccination campaign [62]. Among these 13 USA studies, two were not real-world assessments but rather computer simulations conducted by the same authors, assessing numerous scenarios and operating parameters in order to plan a mass vaccination center [51,52]. 

The remaining two articles reported on experiences from the People’s Republic of China [58] and India [63], but neither was focused on anti-SARS-CoV-2 vaccination. In fact, the first one was based on a vaccination campaign to prevent typhoid fever and meningitis A [58], and the other one on a vaccination campaign with an oral cholera vaccine [63] (Table 1). 

### 3.3. Preparation Needs

To set up an MVC, several important organizational aspects must be considered, and, as a starting point, several studies [53,54,55,56,57,60,61] used the Center for Disease Control and Prevention (CDC) guidelines [11]. These guidelines were mainly developed for a large-scale influenza vaccination campaign and suggested structuring the entire flow in four main steps. The first is orientation: people should be guided by traffic flow personnel when arriving at the center, and they should be screened for potential symptoms. Step two involves staff support in filling forms and assessment of contraindications. Step three is the vaccination; and the fourth step is the post vaccination observation, during which personnel can answer remaining questions, inform about vaccination schedules, and exit. Interestingly, despite the relevance of the vaccination site, only two articles underlined the importance of identifying, inspecting and evaluating the potential site [50,51]. Similarly, only two articles [50,58] analyzed the aspect of the reception and transport of the vaccine: the first considered the mode of transportation and percentage of vaccines that may break or spoil during this process, while the second identified pharmacists and safety officials as professional figures in charge of the process. In detail, the safety officials were responsible for opening, inventorying and transporting vaccines to the mass vaccination center, where pharmacists reconstituted the vaccine.

On the other hand, a larger share of collected reports focused on the human factor of mass vaccination campaigns, as five articles highlighted the importance of preventive identification of the required personnel [49,50,52,58,59], while seven of them [50,55,57,58,59,61] suggested the need for meetings in the period preceding the actual start of vaccine delivery. More precisely, Andress et al. [50] highlighted the importance of identifying consultants, educators and personnel; Jenlink et al. [57] of the updated status of vaccination plans and disease data; and Swift et al. [61] of identifying challenges and opportunities. A total of nine articles [50,51,53,55,57,58,59,61,63] stressed the necessity of personnel training, not only on operating procedures such as vaccination storage and recombination of the vaccine, but also in data entry [53].

The types of equipment necessary to the MVC were detailed by a total of five reports, with recommendations focusing on communication (i.e., phones and fax, two-way radios, communication hardware and software) [50,51,52,55,59], logistical (e.g., portable toilets; wheelchairs, etc.) [50,51], and medical assets (i.e., medical and medical assistant personal protective equipment, staff member vests) that also included items often overlooked such as video/DVD players and video monitors [50,52,55,59]. Additionally, four articles underlined the importance of having registration forms and informational videos in multiple different languages [49,56,57,59]. Interestingly, the collected reports suggest that staff member vests are important not only as part of the personal protective equipment of MVC personnel, but also for allowing the easy and clear identification of their role by patients. 

Moreover, a total of three articles [56,58,63] reported the implementation of a prior and well-structured promotional and educational campaign specifically aimed at the target population. This intervention could contribute to ensuring or improving the success of a mass vaccination campaign by stressing the social and practical importance of the coveted goal of herd immunity.

### 3.4. Layout

All included articles analyzed the MVC location and layout, the latter intended as how it is designed or arranged and its internal organization. In just three studies [60,61,63] the vaccination center was inside a hospital; in contrast, in the majority of them [49,53,54,55,56,57,58,59,62,63], it was in a building not normally used for healthcare purposes, for instance, schools, clubs, shopping malls, factories, stadia, squares, and auditoriums. The areas and rooms needed inside and outside the vaccination center were described in nine studies [49,50,51,52,55,56,59,61,62]; overall, it emerged that they were parking, entrance, registration area, triage, waiting rooms (where people stay both before and after vaccination), educational area, medical evaluation rooms, vaccine storage, vaccination station, post-vaccination area, and exit. An outdoor triage station and/or an observational tent out of the main building were irregularly reported [49,61,62]. Furthermore, an emergency medical tent staffed with paramedics and emergency medical technicians could be placed, even outside of the main building [61]. The importance of signage was stressed in several articles and it was identified as one of the most critical and frustrating aspects of the whole implementation [50,51,52,54,57,59,62]. Not coincidentally, three studies [50,55,61] suggested drawing signs identifying the stations and barrier tape to guide patients through the process, while five articles suggested to objectively show the layout through a site map [51,52,53,55,61]. 

Relative to the internal organization, many articles gave some important advice listed below. While subjects are in the waiting rooms, they can view educational videos and/or read and complete forms on the clipboard provided [49,50,51,52]. One study concluded that in order to facilitate the obtaining of an optimal vaccination rate, the vaccinator should seat between the two seated patients, with a drawer that should stay on the other side of the room ensuring a steady supply of the vaccine [53]. Among the 15 retrieved articles, 4 articles described the flow that patients should follow to best identify available vaccination stations: two of them [49,55] suggested a linear patient flow divided in two lines according to medical history (patients with or without possible contraindications)—patients who have possible complications based on their medical history go to the consultation station; the others sign a consent form and go directly to the vaccination station. The third article [56] described a unidirectional linear flow from the gathering area, multiple stations for eligibility, screening, completion and review form, vaccine administration, and one post-vaccination area. The fourth article [61] split patients in four lanes greeted by a staff member for each lane who directed them to 18 vaccination stations distributed horizontally. The Incident Command Center was described in two articles, and it was responsible for coordinating communications and resources [59,61]. Interestingly, one article [54] described a completely different type of mass vaccination center: a drive-through clinic. This was presented by the authors as an efficient method for delivering, in a quick and safe manner, vaccines to a mass of people, and it was considered extremely important in case of highly contagious infections, like COVID-19. Nevertheless, their internal organization appeared to be similar to the others, and it is composed of arrival, consent hand out lane, consent form fills in lane, vaccination at the point of administration, detour and depart.

### 3.5. Dimensions of Mass Vaccination Center

Four articles indicated the dimensions of the MVC [50,52,54,61]; in more detail, two of them gave the dimensions for each room and its capacity [52,54], the others just the total size [50,61]. Interestingly, the total dimension range varies between 4800 and 105,000 square feet.

### 3.6. Pharmacy Room

Across all the included studies, five [50,55,56,58,63] stressed the presence of a pharmacy room in order to store the vaccine in standardized boxes. Specifically, they described the number of refrigerators and freezers [63], and refrigerated transportation [56,58]. The general recommendation was to put the vaccination stations close together and near the supply box [55]. More details are provided in Table 2.

### 3.7. Restrooms

Restrooms for personnel, such as lunch and break rooms, stocked with food and beverages, were considered as a relevant part of the MVC in three articles [50,60,61]. As general features, they should be large enough in order to guarantee safe accessibility for all the personnel [50]. In order to improve their usability, coordination by an area supervisor was sometimes recommended [60].

### 3.8. Staffing and Medical Procedures

Among all included articles, 11 described the type and/or competences of staff needed in a mass vaccination center [49,50,53,55,56,57,58,59,60,61,63]. In particular, the most frequently reported were physicians, nurses and pharmacists among medical staff, whereas traffic flow personnel, data collection personnel, and volunteers (nursing students, community helpers or school staff for those who implemented the vaccination center in a school) among nonmedical staff. Moreover, in three articles, authors highlighted the importance of human resources managers to supervise the tasks of each staff member and ensure that everyone understood their role [50,53,61].

Regarding the pre-vaccination visit, most of the articles generically referred to a data collector, whereas only three studies specified that the visit was performed by medical staff, including either physicians or nurses [50,55,56]. Regarding the medical staff actually performing the vaccination shot, the majority of the articles generically reported on “vaccinators”, not otherwise specifying their professional requirements. On the contrary, two articles employed the even more vague term of “medical staff”, while only 5 articles out of the 15 we collected clearly stated that vaccinators were nurses [49,56,60,61,63], pharmacists, or nursing and pharmacy students certified to administer intramuscular injections [61]. Conversely, physicians were mainly involved in the post-vaccination tasks, monitoring the acute adverse events following immunizations. The waiting time was usually 15 min [56,57,63] and the room was equipped with emergency medical materials [53].

### 3.9. Timing and Performance

Regarding timing and performance, collected data were quite heterogeneous. Among the 15 retrieved articles, 3 of them [54,60,62] reported the total time needed for the vaccination process, from which a range of 14–30 min for each subject was eventually calculated. On the other hand, two articles [49,52] specifically indicated the targeted time needed for each stage of the vaccination process. On average, triage required 2 min, registration 3 min, receiving educational videos 3–5 min, medical evaluation 2–10 min, and vaccine injection required 2–4 min. In order to have a final tally of how many vaccinations could be done for each vaccination session, five articles specified the number of vaccinations per hour [52,54,55,56,59], with an estimated capacity of around 713 vaccinations per hour. Interestingly, the performance of the drive-through clinic [54] reported a similar result with 12,613 vaccinations, served via 10 drive-through lanes, in 2 days with 12 working hours each.

More in detail, one article [59] reported 640 vaccinations per hour with a staff composed of 36 nurses, 10 recording data electronically, 2 people to greet at the entrance, 5 traffic flow personnel, 15 persons to screen for vaccine eligibility and 2 to maintain real time hourly vaccine counts. In the latter vaccination center, each vaccination session lasted 8 h. Lastly, three articles [53,61,63] reported the number of administered vaccines per vaccinator per hour or per day, from which emerges that the average is about 264 for each vaccination station per day, considering 8 working hours. 

Few of the retrieved articles reported weekly opening days because most of them were simulations of massive vaccination centers or single-day experiences.

## 4. Discussion

Results of our rapid review highlighted the high heterogeneity around the characteristics, layout and management of an MVC. Moreover, only a few articles were retrieved, and almost all of them did not refer to the COVID-19 vaccination campaign, reporting on previous vaccination campaigns that were hardly comparable in terms of targeted population and logistic issues—for example, the necessity to respect all of NPI during all of the vaccination procedures in order to avoid the spread of the pathogen because of the mass gathering represented by the MVC themselves. Interestingly, some of the available guidelines on SARS-CoV-2 vaccination centers (e.g., Quebec Health Ministry, German Committee on the Protection from Biological Agents, or ABAS, but also the Operating Framework of British National Health Service) have clearly recognized such relationship, and the potential shortcomings [12,64,65]. Despite the overall heterogeneity found, some aspects can be considered as a core element of an MVC and, not coincidentally, have been implemented by the aforementioned national guidelines. 

Firstly, in most of the cases, an MVC could be started up in sites that are not originally designed for providing healthcare services. Secondly, an MVC may be only temporarily used, with the aim to centralize as much as possible the vaccination procedures, ensuring high volumes in the shortest time period. In fact, a single vaccination center, instead of multiple locations, might facilitate staff management, supply and avoid surplus or shortages in one of the centers [61]. However, in the case of multiple centers, Asllani et al. suggested to create a network among the MVCs in order to share resources dynamically [51], but such a recommendation has been only acknowledged by Quebec guidelines [12]. In light of this, we suggest a potential definition of an MVC, i.e., a location normally used for non-healthcare-related activities set up for high-volume and high-speed vaccinations during infectious disease emergencies. Examples of mass vaccination sites could include stadiums, exhibition and convention halls, airports, stations, theme parks, museums, and universities or other temporary indoor or outdoor facilities. Particularly in European settings, where an older and aged population may find growing difficulties to reach facilities that are often located in peripheral areas, relatively smaller but widely spread structure may be particularly useful—for example, churches/religious structures and schools [65]. In this regard, a school could be considered a particularly good location for child vaccination because guardians/parents do not need to take time off from work, and this aspect could increase velocity and vaccine acceptance [56,66,67,68], particularly if available vaccines against SARS-CoV-2 will be eventually licensed for children and adolescents [69].

However, as available studies were largely focusing on pathogens other than SARS-CoV-2, some of the aforementioned options may be only partially appropriate. For example, German ABAS has recently stressed the importance of prioritizing facilities where the implementation of SARS-CoV-2 specific NPI was guaranteed through appropriate distancing, ventilation, appropriate access and waiting spaces [64]. 

Another relevant issue is the number, role and type of staff enrolled. Despite the fact that vaccination is a medical procedure, the vast majority of personnel involved were the nonmedical staff. Indeed, the most critical aspects that need to be carefully managed are those related to logistics regarding both supplies and people. Regarding supplies, the most important aspect is quite obviously the availability of a sufficient amount of vaccines, their reception and internal management, including their safe storage, particularly in terms to temperature control. In this regard, COVID-19 vaccines have some specific requirements. Indeed, mRNA-based vaccines require extreme-cold storage conditions, while adenovirus-based vaccines can be stored either in liquid or freeze-dried form, at temperatures (respectively −18 °C and 2–8 °C) that are compatible with a more conventional cold chain [70]. Moreover, the high precision required in vaccine reconstitution necessitates dedicated areas, as well as highly trained personnel. Because these requirements are specific for some of SARS-CoV-2 vaccines, they were not clearly addressed in most of available studies on MVCs; however, quite surprisingly, this significant shortcoming was only irregularly reported in available guidelines. For example, British NHS framework clearly states that a vaccination center must “ensure a sufficient fridge capacity for vaccines, that the areas is secure and there is an area suitable for vaccine preparation” [65], while no specific recommendations are reported by Quebec and German recommendations [12].

Regarding the human factor, the main issues are related to the simultaneous mobilization of large groups of individuals, which only partially could be equated to a mass gathering event. In fact, on one hand, controlling both inside and outside flow through draw signs identifying the stations, the use of barrier tape to guide patients, traffic flow personnel (inside) and personnel from the department of social security (outside) is fundamental especially in managing arrival rate, mode of transportation, point of dispatching and general traffic condition [50,51,52,54,57,59,62]. On the other hand, these people might need health information, reassurance on vaccine safety and efficacy [71], clarification on doubts, and assistance throughout the process [72]. Even though this specific point has been previously stressed by available studies, it has only been scarcely addressed by available recommendations, with potentially severe consequences [71]. In fact, while earlier reports suggested that the COVID-19 vaccination could be well received by the general population [73,74], a growing body of evidence, particularly from USA and Europe, suggests that a large share of the general population may exhibit substantial vaccination hesitance toward COVID-19 vaccination because of paved side effects (as thrombosis) or due to the rapidity by which vaccines have been issued [75,76]. For this reason, some authors suggested using educational videos during the waiting phase before vaccination. In general, clear and frequent communication with the public and staff is crucial for the successful implementation and delivery of immunization clinic operations [72,77,78]. To achieve this goal, duplication of vaccine educational signage and content in other commonly spoken languages is needed. Indeed, it is fundamental to assist people in their specific language in order to guide them throughout the immunization processes. In this regard, some guidelines have somewhat overtaken original reports from scientific literature, pointing towards an extensive use of pictorial representations in order to overcome potentially reduced literacy even in the original vehicular language of migrants and minorities [12,65]. In some cases, onsite translators/interpreters or telephone translation services have been used [79]. Although only four articles dwelled on this specific aspect [49,56,57,59], considering that such reports referred to MVCs located in areas where several languages are spoken (USA, China), and stating that it was specifically addressed by some guidelines, it can be speculated that this potential shortcoming may be widespread, representing a routinary issue not needing to be specifically discussed. Indeed, taking the example of the USA, signage, educational contents for patients, and vaccine educational campaigns were conducted in at least two languages, English and Spanish, as well as potentially several others depending on local community demographics. In particular, one article [56], describing the experience of an MVC in a public school located in Virginia, referred to 121 languages to be taken into consideration. Specifically, information line staff sent communications home in English and Spanish, but with a special message on the envelope in the most common other languages, informing families that this was important information they should have translated. Instead, with fewer languages to consider, Minneapolis (Minnesota) translated materials into Hmong, Somali, and Spanish specific to each school’s population and had interpreters available in MVCs [56]. Nevertheless, nurses, nursing students and generally trained personnel were also involved in helping individuals to fill in the consent form, checking for correctness, and screening for vaccine eligibility. Indeed, two studies used a different approach according to which, based on medical history (subjects with or without contraindications), subjects were immediately directed to vaccination in case of no contra-indications, or to the consultation station in case of contra-indications. In light of this, training personnel is extremely important. Andress et al. [50] clearly reported which type of information should be targeted by which professionals: public health experts to delivery information regarding vaccination plan; veterinarians for describing biological outbreak containment; nurses to demonstrate vaccination procedures; pharmacists to teach about storage and reconstitution of the vaccine. Accordingly, Swift et al. [61] supplied each vaccination station with a job action sheet including specific steps, role and responsibility of each staff member. Additionally, Caum et al. [53] suggested training personnel also in electronic data entry. Indeed, even if electronic data entry might increase the burden in the short term, it highly increases the usability of data collected for estimation of vaccination coverage and other statistics [80]. Nevertheless, ensuring a good quality of electronic data record remains fundamental in the whole process.

Another important aspect found is the layout of the center. Despite the slight differences retrieved among the articles, the most common aspects were entrance, registration, waiting rooms (in many cases with educational video), screening/anamnesis, vaccination room, post vaccination room, and exit. Moreover, a unidirectional linear flow from entrance to exit was the most used, but only in one case where the vaccination stations were positioned horizontally [61]. This is a crucial point to consider when planning the vaccination center, especially in cases of epidemic scenarios with highly contagious and infectious pathogens like SARS-CoV-2. Indeed, to prevent transmission of COVID-19 at MVCs, it will be essential to minimize crowding, ensure physical distancing and separate the different areas. Best practices in spacing clients and minimizing crowding include personnel directing people flow, online and phone appointments and registration, wristbands or ticket number [65]. Moreover, people could wait outside the center for their appointment, evaluating options such as tents and heaters during the winter or inclement weather. The post-immunization waiting area could be a potential location where people, kept under observation for at least 15 min to monitor for immediate vaccine reactions, may be too close together. Therefore, MVC should be organized in order to isolate incoming people from the others and to ensure they are at least 2 m apart at all times with their masks on. All these social distancing rules should be adhered to throughout the model for both safety and privacy issues. Despite the high importance of all these aspects, they only partially emerged from our review because only one article described the COVID-19 vaccination center and all the others referred to other microorganisms with a different infection rate [62]. Another consideration that we can raise from this review is that even the extremely high importance of simultaneously managing a large number of individuals, the size of the center, as well as the need to identify and to inspect in advance the potential site was marginally reported on by a very small number of studies [50,51,54,61]. This aspect could be explained because almost all the included studies reported simulation or single-day experiences. However, although the effectiveness of this approach can be quite limited, simulation offers an excellent and relatively inexpensive opportunity to test alternative scenarios analysis and to develop effective solutions to management problems. Indeed, the simulation outputs visually and numerically show the processing and waiting times, number of cars and people that can be served under different situations. Computer simulations can be considered as pivotal tools in modeling different operational solutions for complications that can occur in any of the critical vaccination phases. Indeed, the high degree of dynamic uncertainties can be forecasted, helping planners to visualize what would happen. Lastly, just one article described the organization and performance of a drive-through mass vaccination center. Even taking into consideration the paucity of evidence, this layout could be considered particularly suitable for the current highly contagious pandemic because it allows a high level of isolation among subjects that do not need to exit their own cars.

### Strengths and Limitations

Before generalizing our results, some strengths and limitations should be addressed. The first strength of our work is the systematic but rapid approach used. Indeed, rapid reviews are an emergent method used to collect, analyze and interpret available evidence. This is especially true nowadays, when the digitalization and the rapidity of evidence production call for a continuous update of available knowledge [81]. In this context, rapid reviews are extremely useful for collecting emerging evidence that can be promptly used by policymakers to inform their decisions, particularly when difficult decisions have to be made in circumstances of emergency, such as the current COVID-19 pandemic. However, despite being systematic in nature, our review was limited to only four databases. Nevertheless, we assessed five databases overcoming the minimum standards (at least two) set by the PRISMA guidelines for systematic reviews. Secondly, we did not strictly focus on COVID-19 MVC; and although it could be considered as a limit of our review, on the contrary, we believe that having also included other mass vaccination campaigns represents an added value to our work. Using this approach, we were as comprehensive as possible, taking other related experiences also into account. As mentioned, only one article on a COVID-19 mass vaccination center was identified. Moreover, a comprehensive description of an MVC planning could also have been limited by the under reporting of many practices that were so obvious, ubiquitous and/or routinely implemented that they were not uniformly and consistently reported. Some examples were the use of MVC signage and educational materials translated into multiple languages reported in just four articles [49,56,57,59], and the conduction of promotional educational campaign aimed at the desired or target population for vaccination that was reported in just three articles. Actually, educational campaigns that are coordinated with public health officials and that include trust brokers are core public health and immunization best practices to widely publicize a mass vaccine clinic before implementation. Thus, even if some aspects are described in only a few articles, it is not possible to conclude that they have not been taken into account for the organization of other MVCs, rather than they have simply often not been reported. Among limitations, we should address the English limitation applied. However, because no articles were removed because of this language limitation, we are confident that our results are not affected by selection bias. Nevertheless, four articles were excluded because we were not able to retrieve the full text [18,19,20,21]. Specifically, the first was published in 1985; the second was a conference paper; the third also lacked an abstract, so it was not possible to make any assessment of the content; the last one evaluated similarities and differences in access and acceptance between vaccinations carried out in an MVC and those in a clinic (information extracted from the abstract). Taking into account all these considerations, we are confident that these exclusions did not bias our results. 

Another significant limitation is represented by the substantial lack of evidences from Western countries other than the USA. This is particularly frustrating because some of the most successful mass campaigns against SARS-CoV-2 were performed in Israel and in high-income countries from Western Europe. In fact, such countries are only limitedly comparable to the USA, not only for the demographics (for example: in European Union, age group 65 years or older encompasses around 20.5% of the total population, compared to 16.2% in the USA) but also in terms of urban planning, with obvious consequences on the availability of adequate and accessible facilities to be converted in MVCs.

Lastly, to this day, the literature concerning this topic is still relatively sparse, allowing us only to draw preliminary conclusions. Moreover, the data mainly referred to simulation and single-day experiences that do not allow us to assess the long-term performance and impact of these mass vaccination centers. Nevertheless, to the best of our knowledge, this is the first review assessing the organization, implementation and performance of mass vaccination centers. In our view, this is an extremely relevant topic both for public health experts and policymakers involved in facing the challenges and threats posed by an infectious pandemic unprecedented in recent human history.

## 5. Conclusions

MVCs are usually acknowledged as the best solution to administer vaccines in the shortest time to the greatest number of people. Our results highlighted an important gap in knowledge because only a very small number of articles was retrieved on the topic. Moreover, these few available articles often under-reported many aspects of MVC organization. The current review answers to the urgency of organizing an MVC during the COVID-19 pandemic, highlighting the most important organizational aspects that should be considered in the planning. Among the others, the most important are the following: the identification of the site; the layout of the center; the identification of the number, role and type of the staff members; the training of the staff; the necessary equipment and vaccines transportation, cold-chain maintenance and storage. However, it should be kept in mind that organizational models might be context-specific based on structural needs or professional availability. Future researches should help better identify the necessary strategies in order to obtain an optimal vaccination rate across the mass vaccination centers, especially on the COVID-19 vaccination.

## Figures and Tables

**Table 1:** General characteristics of the included studies.

Author	Year	Country	Vaccine	Preparation Needs	Layout	Dimension *	Location
Aaby et al. [49]	2004	USA	Smallpox	Not available	Triage station outside the center where linear flow split in two lines according to medical history, registration station, waiting rooms with educational videos, screening station, consultation station (only for people with comorbidities), vaccination station, exit	Not available	School
Andress, et al. [50]	2003	USA	Smallpox	Strategy meetings; visit the potential sites; training involved personnel, staff distinctive vest for personnel	Vaccine storage, triage, screening, education, isolation, vaccination, computer entry, with signs identifying the stations	105,000 square foot	Shopping mall
Asllani et al. [51]	2007	USA	Influenza	Trained personnel, transportation, equipment, retrieve available guidelines, vaccines, visits to the potential sites and investigate the layout	Registration immediately after entrance, waiting rooms with educational videos, medical evaluation rooms, vaccination station, and exit	Not applicable	Computer simulation
Asllani et al. [52]	2007	USA	Influenza	transportation vehicles, medical and assistant equipment such as wheelchairs	Entrance, registration area, waiting rooms with educational videos, vaccination room or medical evaluation area for those with comorbidities, waiting room and exit	Not applicable	Computer simulation
Caum et al. [53]	2013	USA	Influenza	Estimate number of doses needed, ensure that stocks arrived, evaluate which vaccine formulation to offer, understand characteristics of target population (if any special needs), training personnel also in electronic data entry	The vaccinator sat between the 2 seated patients to facilitate rapid access to patients. Drawer was on the other side of the room ensuring a setady supply of vaccine. For each patient’s seat there was a data collector	Not available	School
Gupta et al. [54]	2009	USA	Influenza	Not available	Arrival, consent hand out lane, consent form filled in lane, vaccination at the point of dispense, detour and depart	Vehicle gap length 12 feet, length of the consent form lane 950 feet, length of vaccination lane 50 feet	Drive-through clinic at a stadium
Ha et al. [55]	2014	USA	Influenza	Standardized training for personnel, meeting, staff distinctive vest for personnel	Linear flow split in two lines according to medical history; with signs identifying the stations	Not available	Auditorium
Jenlink et al. [56]	2009	USA	Influenza for children	Promotional campaign to the population only when sure about vaccine supply. One lot per day for each center in order to print the lot number on all the form and save time. Determine target population for the vaccine. Secure supply	Unidirectional linear flow from gathering area, multiple stations for eligiblity, screening, completition and review form, and perform vaccination; and 1 post-vaccination area	Not available	School
Jenlink et al. [57]	2009	USA	Influenza for adults	Preparation meeting during the summer in order to know the vaccination plan and flu update	Not available	Not available	Clinics with school nurses
Kar et al. [58]	2011	India	Cholera	Training for staff	Screening, verbal consent, vaccination station, registration station, waiting room, issued vaccination cards, collected remaining vaccine vials and waste at the end of each session, and brought waste back to the designated health facility	Not available	School and local clubs
Phillips et al. [59]	2004	USA	Influenza	Staff meeting one week before to ensure availability of administrative and clinical supplies, distribute staffing schedules, and order staff refreshments. A mandatory meeting for all staff involved was planned the day before the mass vaccination day	Incident Command Center, staff accommodations, restrooms, entrance and exits, parking and proposed traffic flow with signs and marking corridors	Not available	School
Porter et al. [60]	2009	USA	Influenza	A good reservation system in plan, scanner, personnel	Not available	Not available	Health department location
Swift et al. [61]	2011–2015	USA	Influenza	Staff meetings to identify challenges and opportunities. Training for staff	Entrance in the midway along the long side of the tent, 4 lines with traffic flow personnel who directed to 18 horizontal vaccination stations. Vaccination station, documentation stations and exit. The vaccination tent also contained the incident command center. Next to the vaccination tent there was an emergency medical tent	40 × 120 feet	Outdoor tent clinic outside the hospital
Wheeler et al. [62]	2021	USA	COVID-19	A phone app and web-site appointment systems	Parking, check-in station, waiting area, recovery and observational tent	Not available	Disneyland parking
Yang et al. [62]	2003	China	Typhoid fever and meningitis A	Promotional campaign, training for staff, simulation with a sub sample	Multiple vaccination centers (107), one for each cluster	Not available	School, health facility, factory or locations such as intersections and squares

* Dimension of the building. USA: United States of America.

**Table 2:** Detailed description of the rooms, role and performance of the mass vaccination centers presented in the included studies.

Author	Pharmacy Room	Restrooms	Waiting Room Capacity and Management	Staffing	Medical Procedures	Timing/Performance	Others	Map
Aaby et al. [49]	Vaccines stored at the logistic hub at Hechi CDC: a 3 × 12 m^2^ room equipped with 8 refrigerators and 1 freezer	Not available	Not available	Staff number needed for each stage: triage: 5; registration:8; education: 8; screening: 9; consultation: 6; vaccination: 16	Injection performed by nurses. Medical history collectors not available	Time for each stage: triage: 2:18; registration: 2:43; education: 31:23; screening: 16:77; vaccination: 8:87; total in system 60:02 Working time: 2:30 p.m.–3 p.m.	Not available	No
Andress, et al. [50]	Forecasted without details	Large enough in order to guarantee accessibility for all	Not forecasted	140 in total, including human resources manager, translators, security and nurses	Medical history collection and vaccine injection performed by medical staff (nurses and physicians)	104 vaccinations in 2 h (it was a 2-h exercise)	Not available	No
Asllani et al. [51]	Not available	Not available	Not available	Not available	Not available	50,000 vaccinations in 3 days	Creation of a network among the mass vaccination centers in order to share resources dynamically	Yes
Asllani et al. [52]	Not available	Not available	Not available	Not available	Not available	700 vaccinations per hour; vaccination session lasted 24 h. Time for each stage for person: registration 30–90 s, registration form 2 min, video watch 3–5 min, medical evaluation 2–10 min, vaccine administration 2–4 min	Not available	Yes
Caum et al. [53]	Not available	Not available	Stores of emergency medical materials; capacity not available	1 vaccinator, 1 drawer, 1 data collector with a range of vaccination stations up to 14. Presence of a human resources manager to supervise the tasks of each staff ensuring that everyone understood their role	Data collectors interviewed the students. Injection performed by vaccinator (not otherwise specified)	32–45 people for each vaccination station per hour; vaccination session lasted 1:30 (1:05–2:35 p.m.). In total 52 people in 54 min	Preference of electronic data entry	Yes
Gupta et al. [54]	Not available	Not available	Not available	Not available	Consent form workers who distributed and received the filled-out consent forms. Injection performed by medical workers	7732 vaccinations in a 12 h (7 a.m.–7 p.m.); 12,613 served via 10 drive-through lanes after two days. Time for each person: 27.4+/−0.8 min	Not available	No
Ha et al. [55]	Store vaccines in standardized boxes, placed where trained staff exactly know. Vaccination stations placed closer together and near the supply box	Not available	Not available	40 in total	Medical history collected by nurses or healthcare providers. Injectors not available	4500 vaccinations in 6 h (time for each vaccination session). Working days: 3	Not available	Yes
Jenlink et al. [56]	Strict regulation of thermometers. Attention to the refrigerated transportation	Not available	15 min of observation; capacity not available	Nurses paid and on a voluntary basis (nursing students). For each vaccinator 4 nonmedical staff useful as greeters, traffic direction, form review, and supply runners. School staff to obtain consent for vaccination	1 to 10 rooms each with 5 to 6 nurses for screening and checking the form and vaccinate	2500 s doses in 3 h (time for vaccination session). Time for each person: 30 min	School was a good location for child vaccination because guardians/parents did not need to take time off from work	No
Jenlink et al. [57]	Not available	Not available	15 min of observation; capacity not available	School staff to obtain consent for vaccination. Nurses to check the consent form	Not available	11,200 vaccinations in 5 h for the 9 clinics. 100 appointments for every 5–8 min settled by the call center dedicated. Working days: 3 weeks, during the evening or on Saturday	Not available	No
Kar et al. [58]	Not available	Not available	Not available	395 health workers/volunteers organized in team	Not available	Working time: 7:00 a.m.–5:00 p.m. for 3 consecutive days in each round from 5 May to 4 June 2011 (in total 15 working days)	The walk-in cooler temperature was monitored and maintained between +2 to +8C	No
Phillips et al. [59]	Not available	Not available	Not available	36 nurses, 10 personals to record electronically data, 2 persons to greet at the entrance, 5 traffic flow personnel, 15 persons to screen for vaccine eligibility, 2 persons to maintain real time hourly vaccine counts	Not available	640 vaccinations per hour; each vaccination session lasted 8 h (8:20 a.m.–5 p.m.). Working days: 2 consecutive Saturdays	The fire department provided a basic life support ambulance on sire, and voluntary companies provided refreshments from a fully equipped service vehicle	No
Porter et al. [60]	Not available	Breaks and lunches coordinated by area supervisors	Not available	1 vaccine preparator for 4 vaccinators, 133 physicians, 43 vaccinators, 11 vaccine preparer, 77 administrative staff	Medical history collected by staff. Injection performed by nurses	7889 vaccinations in 9.5 h (time for each vaccination session). Time for each person: 15 min	Not available	No
Swift et al. [61]	Not available	A staff break room stocked with snacks and beverages	Tent located adjacent to the vaccination clinic; capacity not available	Leaders from occupational health, nurses, pharmacists, student health, supply management, human resources, safety and event officers	Injection performed by nurses or pharmacists or nursing or pharmacy students certified to administer intramuscular injections. Medical history collectors not available	12,850 with 37.1 vaccines per vaccinator/hour. Each vaccination session lasted 8 h (10 a.m.–4 p.m.). Working days: 1–2 days/year per 5 years in total	Each vaccination station was supplied with a flag system allowing vaccinators to raise color-coded flags Job action sheet were provided to the staff. Prefer one location in order to facilitate staff management, supply and avoid surplus or shortages in one of the centers	Yes
Wheeler et al. [62]	Not available	Not available	Tent with medical staff; capacity not available	Not available	Not available	7500–8000 vaccinations for each vaccination session. Time for each person: 30 min	Not available	No
Yang et al. [62]	Not available	Not available	15 min of observation monitored by physicians; capacity not available	30 physicians, 43 nurses, 24 other health workers and 9 nonhealth workers to record data, 78 community helpers to facilitate the process. Each cluster was provided by a team based on one physician, one nurse, one recorder and one community helper	Injection performed by nurses. Medical history collectors not available	200 vaccinations for each cluster per day. Working days: 31, from 8 April to 12 May 2003	Each vaccination center administered only one vaccine	No

## Data Availability

All data are presented in the current manuscript (text, tables, and Supplementary Materials).

## Supplementary Materials

The following are available online at https://www.mdpi.com/article/10.3390/vaccines9060574/s1, Table S1: Search strategy developed in each database; Table S2: Articles assessed in full and excluded with reasons.
