# Supplementary material for: Challenges and Opportunities of Mass Vaccination Centers in COVID-19 Times: A Rapid Review of Literature
Source: Vaccines (Basel). 2021 Jun 1;9(6):574. doi: 10.3390/vaccines9060574 (PMC8230199; doi:10.3390/vaccines9060574)
Supplement: Supplementary file 1 [file vaccines-09-00574-s001.zip › vaccines-1223713-SI.pdf]

Table S1. Search strategy developed in each database.

DATABASE	QUERY
PubMed/Medline	((“Organization and Administration” [MeSH Terms] OR “Health Planning Organizations” [MeSH Terms] OR “process assessment, health care” [MeSH Terms] OR “health performance” [Title/Abstract] OR “health management” [Title/Abstract] OR “healthcare performance” [Title/Abstract] OR “healthcare organization” [Title/Abstract] OR “healthcare management” [Title/Abstract])) AND (“Mass Vaccination” [MeSH Terms] OR “Mass Vaccination” [Title/Abstract])) AND (english[Filter])
Scopus	(TITLE (“mass vaccination”)) AND (TITLE-ABS-KEY (organization OR planning OR management))
Google Scholar	“mass vaccination centers” OR “mass vaccination center” OR “mass vaccination centre” OR “mass vaccination centres”
medRxiv	Or full text or abstract or title “mass vaccination”
EMBASE	"Mass immunization" AND ("organization and management" OR "health care planning" OR "health care facilities and Services" OR "health care personnel management")

Table S2. Articles assessed in full and excluded with reasons.

References	n. studies	Reasons
Fontanesi, J., et al. 2006 [14]; Nolan, P. et al 2004 [15]; Jarrett, S. W. et al. 1985 [16]; Schindler, J. V. 2008 [17]	4	Full-text not available
Kuehnert, P. 2010 [27]; Lee, T. H. et al. 2021 [28]; Meyer, D., M. et al. 2018 [29]; Velimirovic, B. et al. 1981 [30]	4	General consideration about MVC
Gerber, R. et al. 2007 [31]; Giot, J. L. et al. 2003 [32]	2	Legislative aspects of vaccination
Arthur, B. C. et al. 2015 [33]; Cho, B. H. et al. 2011 [34]	2	Cost analysis of vaccination clinics
Garske, T. et al. 2014 [35]; Porco, T. C. et al. 2004 [36]	2	Model predictions to transmission intensity of infection
Matteson, L. M. et al. 2006[37]; Nicoll, A. et al. 2010[38]; Rambhia, K. J. Et al. 2010 [39]; Rebmann, T., T. e al. 2015 [40]; Rebmann, T. et al. 2015 [41]	5	Development of procedure in case of pandemia
Privor-Dumm, L. et al. 2020 [42]; Sarma, H. et al. 2019 [43]; Schwartz, B. et al. 2006 [44]	3	Analysis of vaccine campaign
Dervaux, B. et al. 2003 [18]; Goralnick, E. et al. 2021 [19]; Ha, C. et al. 2016 [20]; Iacobucci, G. et al. 2020 [21]; Olmsted, S. et al. 2006 [22]; Savitz, S. et al. 2007 [23]; Sim, F. et al. 2021 [24]; Sutter, R. W. et al. 2006 [25]; Szilagyi, P. G. et al. 2003 [26]	9	Several different aspects non-related with the organization of a MVC
